# Comparative Genomic Mapping Implicates LRRK2 for Intellectual Disability and Autism at 12q12, and HDHD1, as Well as PNPLA4, for X-Linked Intellectual Disability at Xp22.31

**DOI:** 10.3390/jcm9010274

**Published:** 2020-01-19

**Authors:** Jonathan D. J. Labonne, Terri M. Driessen, Marvin E. Harris, Il-Keun Kong, Soumia Brakta, John Theisen, Modibo Sangare, Lawrence C. Layman, Cheol-Hee Kim, Janghoo Lim, Hyung-Goo Kim

**Affiliations:** 1 Section of Reproductive Endocrinology, Infertility & Genetics, Department of Obstetrics & Gynecology, Augusta University, Augusta, GA 30912, USAMHARRIS3@augusta.edu (M.E.H.); sbrakta@augusta.edu (S.B.); jtheisen@augusta.edu (J.T.); lalayman@augusta.edu (L.C.L.); 2 Department of Genetics, Yale University, New Haven, CT 06510, USA; terri.driessen@yale.edu (T.M.D.); janghoo.lim@yale.edu (J.L.); 3 Department of Animal Science, Division of Applied Life Science (BK21plus), Institute of Agriculture and Life Science, Gyeongsang National University, Jinju 52828, Korea; ikong7900@gmail.com; 4 Faculty of Medicine and Odontostomatology (FMOS), University of Sciences, Techniques and Technologies of Bamako (USTTB), Bamako, Mali; mouadib@gwmail.gwu.edu; 5 Department of Neuroscience and Regenerative Medicine, Augusta University, Augusta, GA 30912, USA; 6 Department of Biology, Chungnam National University, Daejeon 34134, Korea; zebrakim@cnu.ac.kr; 7 Department of Neuroscience, Program in Cellular Neuroscience, Neurodegeneration and Repair, Yale Stem Cell Center, Yale University, New Haven, CT 06510, USA; 8 Neurological Disorders Research Center, Qatar Biomedical Research Institute, Hamad Bin Khalifa University, Doha P.O. Box 34110, Qatar

**Keywords:** microdeletion, LRRK2, autism, HDHD1, PNPLA4, intellectual disability, parkinsonism, XLID, 12q12, Xp22.31

## Abstract

We report a genomic and phenotypic delineation for two chromosome regions with candidate genes for syndromic intellectual disability at 12q12 and Xp22.31, segregating independently in one family with four affected members. Fine mapping of three affected members, along with six unreported small informative CNVs, narrowed down the candidate chromosomal interval to one gene *LRRK2* at 12q12. Expression studies revealed high levels of *LRRK2* transcripts in the whole human brain, cerebral cortex and hippocampus. RT-qPCR assays revealed that *LRRK2* transcripts were dramatically reduced in our microdeletion patient DGDP289A compared to his healthy grandfather with no deletion. The decreased expression of LRRK2 may affect protein–protein interactions between LRRK2 and its binding partners, of which eight have previously been linked to intellectual disability. These findings corroborate with a role for LRRK2 in cognitive development, and, thus, we propose that intellectual disability and autism, displayed in the 12q12 microdeletions, are likely caused by *LRRK2*. Using another affected member, DGDP289B, with a microdeletion at Xp22.31, in this family, we performed the genomic and clinical delineation with six published and nine unreported cases. We propose *HDHD1* and *PNPLA4* for X-linked intellectual disability in this region, since their high transcript levels in the human brain substantiate their role in intellectual functioning.

## 1. Introduction

Comparative deletion mapping is a powerful strategy to narrow down chromosomal regions of interest to identify underlying disease-causing gene(s) [1,2,3,4,5,6,7,8]. With the availability of high-resolution microarray technology, an increasing number of pathogenic copy number variations (CNVs) responsible for recognizable syndromes are being discovered [9,10,11]. These microdeletion and microduplication syndromes are often associated with intellectual disability, autism spectrum disorders, and multiple congenital anomalies [9,11,12].

Chromosome interval 12q12 is 8.2 Mb in size, containing 28 annotated genes. Interstitial deletions of this region are rare, having only been reported in six patients to our knowledge [13,14,15,16,17,18]. Individuals with 12q12 interstitial microdeletions display a number of clinical features, including psychomotor retardation, craniofacial anomalies, as well as limb and genital anomalies [13,14,15]. Genomic and clinical delineation has enabled the dissection of this region, allowing for the identification of intellectual disability and craniofacial candidate genes *YAF2* (MIM 607534) and *PRICKLE1* (MIM 608500), while haploinsufficiency of NELL2 (MIM 602320) and DBX2 are suggested to be the cause of psychomotor delay and motor delay, respectively [13,14,15].

CNVs at Xp22.31 are frequently associated with X-linked ichthyosis and intellectual disability, particularly when the CNVs encompass *STS* (MIM 300747) and *VCX3A* (MIM 300533) [19,20,21,22]. The *STS* gene located at Xp22.31 has been shown to be responsible for X-linked ichthyosis, a disorder characterized by dry, thickened, and scaly skin [23,24]. The Xp22.31 segment harbors a dozen annotated genes, including several members of the *VCX* gene family, including *VCX3A* (variable charge, X-linked 3A). The *VCX* families have a high degree of sequence similarity and members of this multi-gene family are known to be ubiquitously expressed in human tissues [20,25].

Here, we report a genomic and clinical delineation at 12q12 and Xp22.31 to identify causative genes for intellectual disability. A total of 14 unpublished and five published CNV cases at 12q12 were used for this comparative genomic mapping study.

## 2. Clinical Report

**Figure 1:**
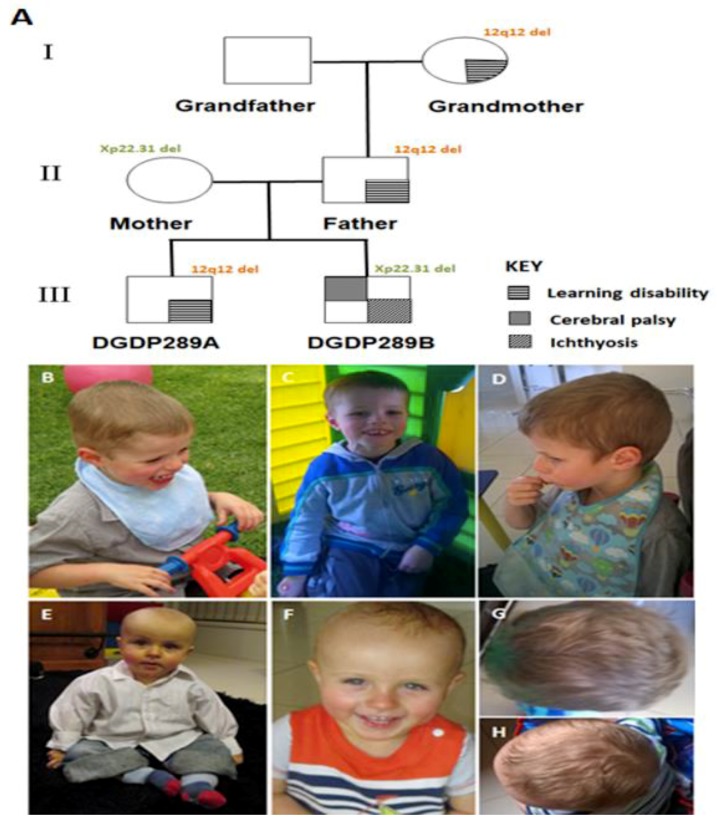
Pedigree and photographs of patients. (**A**) Learning disability of DGDP289A is segregated with 12q12 microdeletion in three generations. The Xp22.31 microdeletion in DGDP289B was inherited from his mother. While the mother is a healthy carrier of this deletion, her son displays ichthyosis and cerebral palsy. Patient DGDP289A has a broad and flattened forehead, as shown by pictures taken at 40 (**B**) and 41 months (**C**). (**D**) Lateral facial view of DGDP289A. DGDP289B displays notable frontal bossing, as shown by pictures taken at 11 months (**E**) and 19 months (**F**). The head shape of DGDP289A (**G**), as well as DGDP289B (**H**) from above, are also displayed.

The proband DGDP298A (Figure 1A) is presently a nine year old boy born to Australian Caucasian parents, the mother currently being 38 years old and the father 42 years old. The pregnancy was complicated by pregnancy-induced hypertension, and the birth was by C-section (birth weight 3.05 kg). The infant had a right undescended testis which was later repaired. At the age of eight months, the proband could only babble and made no progress with words. The sitting and crawling milestones were reached at the age of nine and 18 months, respectively. A microarray at 16 months revealed a 12q12 microdeletion. MRI and fragile X testing at 18 months were normal.

At age 1^7/12^, the proband displayed global developmental delay and mild failure to thrive—weight (3rd), length (10th), and head circumference (75th) percentiles. Vision and hearing were normal. At age two, gross and fine motor delays and motor planning difficulties were blatant. Appearing right-handed, he could scribble, but had not started shape sorting, construction, or completing basic inset puzzles. He had echolalia, but did not sing along with nursery rhymes. The infant showed minimal comprehension and displayed some eye contact. When responding to commands, nodding, pointing and gesturing were observed. However, his responses were uncoordinated and the boy did not consistently respond to his name being called. The infant walked at 28 months and enjoyed transport toys, blocks, books, TV, and music. His play was mostly exploratory to functional and he did not use symbolic, animative or role-play. He was clingy with his mother and observed play when with his peers. Significant communication disorder in expressive, receptive, and pragmatic areas were observed. His social interaction was limited, and he displayed unusual behavioral patterns. His features were most consistent with a pervasive developmental disorder not otherwise specified (PDD-NOS). A score of >30 was obtained on the Childhood Autism Scale (CARS) and in the Autism Spectrum range on the Autism Diagnostic Observational Schedule (ADOS).

At 3^5/12^ years, there were concerns with his comprehension, intelligibility of speech, ability to attend to tasks, and lack of awareness of saliva control. The child started a program to improve saliva control, oral language, and speech production. He engaged in reciprocal play (Figure 1B), and used appropriate verbal and non-verbal control. The proband could sustain on-task behavior for 2–3 min and accept re-direction to complete tasks. On the pre-school language scale, 5th edition (PLS-5), he scored 50 on receptive oral language, 51 on expressive oral language, and 50 on total language composite, all well below average. His forehead appeared broad and flattened (Figure 1B–D).

At age five, the child struggled to follow basic instructions and spoke only a few words. He displayed anxiety, impaired social skills, poor attention, and constant motion. The boy could not dress himself and was not toilet trained. He fell frequently and could not perform one foot activities (kicking and hopping). His father, who struggles in mathematics and suffers from depression, has the same 12q12 microdeletion, as does the paternal grandmother, who was late walking and struggles to move her hand (Figure 1A). She has had life-long learning difficulties and struggles to say some words.

DGDP289B (Figure 1A), the younger brother by two years of DGDP289A, was admitted to the Children’s Hospital at two months of age for failure to thrive. He had ichthyosis due to the deletion of STS, but upon examination there were no apparent concerns about developmental delay. At seven months, the baby weighed 6.01 kg (<3rd percentile). His length was 65.5 cm (10–25th percentile), while his head circumference was 42.6 cm (10th percentile). He also displayed notable frontal bossing (Figure 1E,F,H). At seven months, the infant was not able to roll from front to back or sit, and had limited neck control. He had dry skin, but no lesions or breakdown of the skin. His testes were palpable bilaterally in the scrotum. The toddler did not grasp or reach for toys and often had unusual hand posturing, holding his arms in an internally rotated and extended position. He held his legs in a scissored extended posture and, when standing, did not take much weight and collapsed. However, his muscle tone in all limbs was normal, and he was not hyperreflexic. DGDP289B was also examined by a physiotherapist and a pediatrician, who noted both mild developmental and speech delays. The infant had some feeding difficulties and displayed bronchiolitis. His mother had concerns about plagiocephaly and noted early right-hand preference. Microarray at nine months revealed a microdeletion at Xp22.31 with no chromosome 12 anomaly. At age one, a videofluroscopy was performed by a speech pathologist, during which he participated cooperatively. He presented with delayed swallowing and an aspiration risk. DGDP289B walked at 32 months, and at 36 months displayed gross motor delay. According to his neurologist, his clinical features resembled cerebral palsy. His healthy mother has the same Xp22.31 microdeletion, with no 12q12 genomic deletion.

## 3. Materials and Methods

### 3.1. Cell Culture

Blood obtained from patients DGDP289A, DGDP289B and other family members (Figure 1A) was used to isolate lymphocytes by density gradient centrifugation. From patient’s whole blood samples, lymphocytes were isolated using density gradient centrifugation. Ten mL of the blood sample was layered on top of the 5 mL of Lymphocyte Separation Medium (Cellgro, Manassas, VA, USA) in 15 mL tubes, and the tubes were centrifuged at 400× *g* for 30 min. From the tubes, the lymphocyte layer in the middle was carefully removed and washed in PBS (Fisher, Pittsburgh, PA, USA) twice at 300× *g* for 10 min. Then, the lymphocytes were immortalized by adding cyclosporine A (Sigma, St. Louis, MO, USA) and Epstein–Barr virus (EBV) to the cell pellet, and maintained in RPMI (Hyclone, South Logan, Australia) containing 20% fetal bovine serum (Atlas Biologicals, Fort Collins, CO, USA), 1% l-glutamine (Corning, Corning, NY, USA), and 1% penicillin/streptomycin/amphotericin B solution (Corning, Corning, NY, USA) [26].

### 3.2. Genomic DNA Isolation

Genomic DNA was extracted from blood samples using a standard phenol-chloroform method. [27]. 

### 3.3. Microarray

Microarray analysis was performed using the Illumina HumanCytoSNP-12 v2.1 (Illumina, San Diego, CA, USA) in order to detect pathogenic copy number variations (CNVs).

### 3.4. Quantitative PCR (qPCR) and RT-qPCR

Primers targeting *LRRK2* exon 2 and *HDHD1* 3′-UTR were used to investigate inheritance of the microdeletions at 12q12 and Xp22.31, respectively. We designed primers against the genic and non-genic regions around the boundaries of both microdeletions to determine the location of the proximal as well as the distal deletion breakpoints (Table S1). Primers from non-repetitive regions targeting *PNPLA4* 3′-UTR, *SLC2A13* exon 2, *CNTN1* exon 4, and *MUC19* exon 7 were also designed for RT-qPCR. Total RNA was isolated from LCLs using the RNeasy Plus Mini kit (Qiagen, Germantown, MD, USA) following the manufacturer’s instructions. One microgram of total RNA from the whole human brain, fetal brain, hippocampus, cerebellum, cerebral cortex, heart, liver, lung, and skeletal muscle was purchased from Clontech (Mountain View, CA, USA). Details of the ages of the individuals from whom the total RNA samples originate have been described previously [7]. Synthesis of cDNA was performed using the RevertAid First cDNA Synthesis Kit (Thermo Scientific, Waltham, MA, USA). Real-Time PCR was carried out using 2 µL cDNA, 2.5 µM primer and 10 µL FastStart DNA Green Master (Roche, Indianapolis, IN, USA) in a 20 µL reaction volume. Samples were run in triplicates and standard deviations were calculated from 2–3 independent experiments. The ΔΔct method was used to determine relative quantification of copy number (qPCR) and transcripts levels (RT-qPCR) [28].

### 3.5. Protein Network Analysis

An intellectual disability gene list was assembled from a recently published review and two independently curated databases: DISEASES and MalaCards [29,30]. The genes of interest were compared to known LRRK2-interacting proteins documented in the Biological General Repository for Interaction Datasets (BioGRID) [31]. In order to visualize physical and genetic interactions between LRRK2 and intellectual disability genes, as well as among the intellectual disability genes themselves, the search tool for recurring instances of neighboring genes (STRING) was used but limited to interactions derived from experimentation, co-expression, or database mining [32]. Two direct physical interactions listed on BioGRID were not visualized on the STRING network, and were added manually after the production of the STRING network with relevant citations next to the edges [33,34,35]. Protein and disease names with the Online Mendelian Inheritence of Man phenotype ID can be found in Table 1. 

The built-in STRING enrichment analysis for gene ontology and KEGG pathways was utilized to identify notable cellular components or biological pathways that proteins of interest are localized to. A similar analysis in ToppCluster and NIH DAVID was used to replicate, and verify, STRING findings [39,40].

## 4. Results

### 4.1. Comparative Deletion Mapping at 12q12

**Figure 2:**
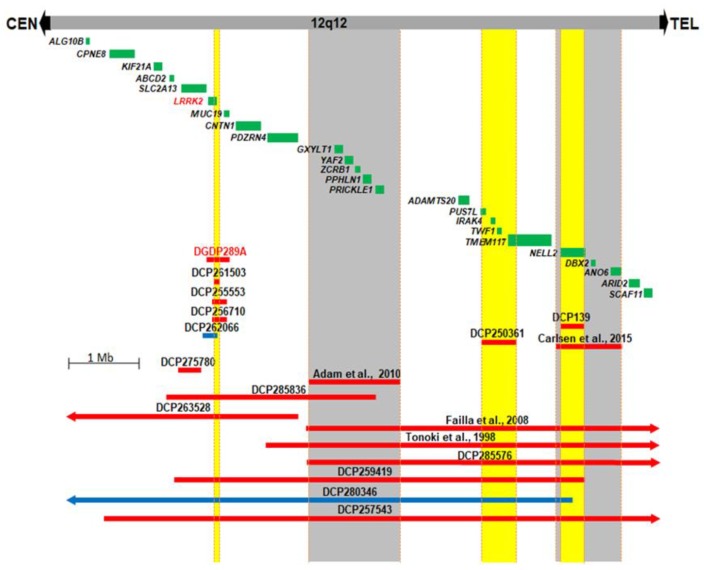
Comparative deletion mapping of CNVs at 12q12. Twenty-four genes residing in this 8.2 Mb chromosomal region are displayed. The microdeletion in our patient DGDP289A, in addition to thirteen unpublished DECIPHER cases, are displayed. The four published cases are also shown across this interval [13,14,15,18]. DGDP289A has a microdeletion involving only *LRRK2* and *MUC19*. CNV mapping with three microdeletions and one microduplication from DECIPHER database, along with DGDP289A, suggest *LRRK2* as a likely candidate gene. Microdeletions are represented by red bars, while microduplications are in blue. The four chromosomal segments with gene deletions, likely producing clinical features such as developmental delay/intellectual disability and autism, are highlighted in yellow (new candidate gene regions) and in gray (reported candidate region). We cannot exclude the possibility that the syndromic intellectual disability in the DCP250361 of the third segment is caused by a position effect of a gene in the fourth candidate region.

We compared the 12q12 microdeletion in our patient DGDP289A with five CNVs (DCP261503, DCP255553, DCP256710, DCP262066, DCP275780) described in the DECIPHER database [41] (Figure 2). The candidate region, encompassing three genes, *SLC2A13*, *LRRK2*, and *MUC19*, was narrowed down to *LRRK2*. In addition to the microdeletion and clinical phenotype in DGP289A, two other DECIPHER cases (DCP250361 and DCP139) and two reported cases suggest a total of four 12q12 chromosomal segments which are likely involved in the clinical features of developmental delay/intellectual disability and craniofacial anomalies (Figure 2) [13,14]. Four chromosomal segments highlighted in yellow or gray (Figure 2), each of which are likely involved in clinical features of developmental delay/intellectual disability and craniofacial anomalies, were identified. The first segment encompasses *LRRK2*, the second segment, a previously published microdeletion [14], includes five genes, *GXYLT1*, *YAF2*, *ZCRB1*, *PPHLN1* and *PRICKLE1*, the third region (DECIPHER case DCP250361) contains deletions of *PUS7L*, *IRAK4*, *TWF1* and *TMEM117*, and, in the fourth segment, *NELL2* is deleted [13] (Figure 2).

### 4.2. Comparative Deletion Mapping at Xp22.31 

**Figure 3:**
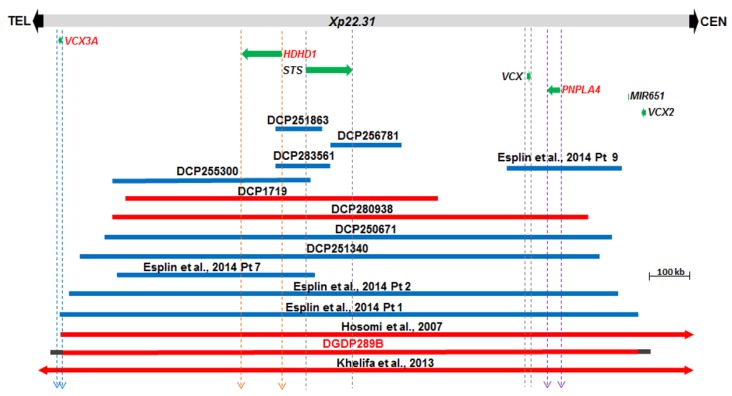
Comparative deletion mapping of CNVs at Xp22.31. The genes proximal and distal to *STS* are displayed. The microdeletion in our patient DGDP289B encompasses at least *HDHD1*, *STS*, *VCX*, *PNPLA4* and *MIR651*. The distal breakpoint lies either within or in the immediate telomeric vicinity of *VCX3A*, while the proximal breakpoint resides around *VCX2*. Three candidate genes, *VCX3A*, *HDHD1*, and *PNPLA4*, are in red letters. A total of eight additional cases were obtained from the DECIPHER (DCP) databases and are included above. Previously published cases include the microduplications in four patients reported in Esplin et al. and two additional microdeletions reported in Hosomi et al., and Ben Khelifa et al. [19,42,43]. Microdeletions are depicted by red bars, while microduplications are represented in blue. Gray horizontal bars at both ends of DGDP289B indicate the putative locations of the proximal and distal deletion breakpoints.

The Xp22.31 microdeletion in the proband’s brother, DGDP289B was compared to 14 CNVs including eight cases derived from the DECIPHER database [41]. The microduplication in DCP256781 involves *STS* only (Figure 3). Four CNVs (DCP251863, DCP283561, DCP255300, and DCP1719) encompass *HDHD1* and *STS* only, while the microduplication in Patient 9 of Esplin et al. spans *VCX* and *PNPLA4* only [42]. Four larger CNVs (DCP280938, DCP250671, DCP251340 and Patient 2 of Esplin et al. include *HDHD1*, *STS*, *VCX* and *PNPLA4* only [42]. The microdeletion in Patient 1 of Esplin et al. truncates *VCX3A* and spans the four aforementioned genes [42]. The CNVs in patients of Hosomi et al. and Ben Khelifa et al. span at least *VCX3A*, *HDHD1*, *STS*, *VCX*, *PNPLA4*, *VCX2* and microRNA *MIR651* [19,43]. The microdeletion in DGDP289B also includes *MIR651*. However, due to the repetitive nature of sequences around the *VCX* genes, we do not know whether *VCX3A* and *VCX2* are also included in the microdeletion in DGDP289B.

### 4.3. Microarray Analysis of DGDP289A and DGDP289B

A 328 kb minimal deletion at 12q12 (chr12: 40,187,072–40,515,212 GRCh38/hg38) was identified in patient DGDP289A by microarray analysis. The genes involved in the microdeletion include *LRRK2* and *MUC19* (Figure 2 and Table 1). Patient DGDP289B did not have the 12q12 genomic deletion but possessed a ~1.6 Mb microdeletion at Xp22.31 (chrX: 6,598,694–8,163,401, GRCh38/hg38). The genes encompassed in the Xp22.31 microdeletion include, *HDHD1*, *STS*, *VCX* and *PNPLA4* (Figure 3 and Table 2).

### 4.4. LRRK2 Transcript Levels Were Significantly Reduced in Patient DGDP289A

**Figure 4:**
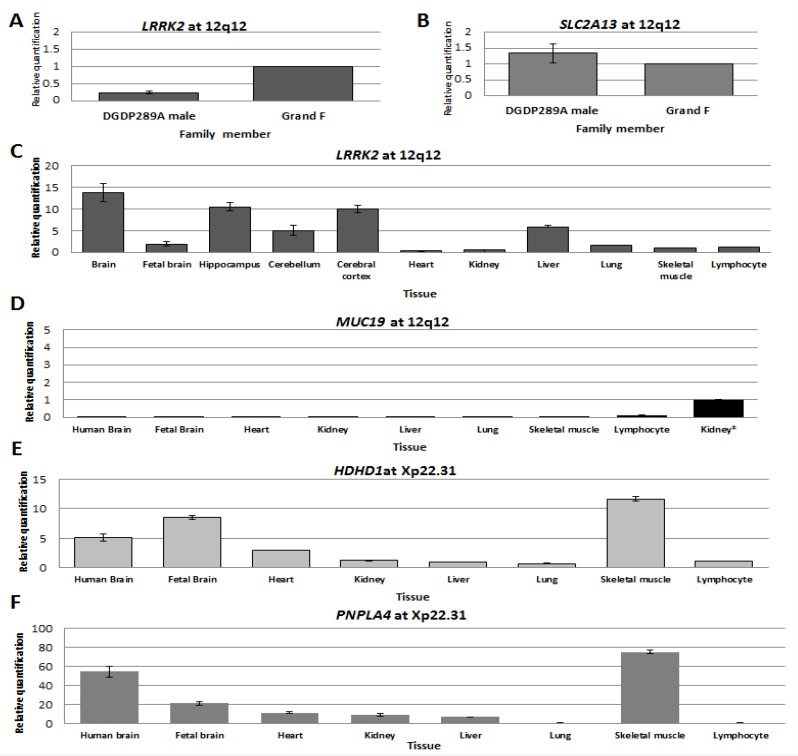
Transcript levels of genes at 12q12 and Xp22.31. Expression levels of *LRRK2* (**A**) and *SLC2A13* (**B**) in LCLs established from patient DGDP289A and his paternal grandfather. (**C**–**F**) Transcript levels of *LRRK2*, *MUC19*, *HDHD1* and *PNPLA4* in the brain and other human tissues were determined by RT-qPCR. (**C**) Transcript levels of *LRRK2* are high in the whole human brain, hippocampus, and cerebral cortex. There is moderate expression in cerebellum and liver. (**D**) Low levels of *MUC19* transcripts were only detected in lymphocytes. The level of *LRRK2* transcripts in kidney was used for relative comparison because of the very low abundance of *MUC19* transcripts. (**E**) High levels of *HDHD1* transcripts were detected in whole the human brain, fetal brain and skeletal muscle. Low abundance of transcripts was observed in the kidney, liver, lung and lymphocyte. (**F**) *PNPLA4* transcripts were very high in the whole human brain and skeletal muscle. Relatively high levels of transcript were also observed in the fetal brain.

RT-qPCR assays revealed that *LRRK2* transcripts were dramatically reduced in DGDP289A compared to his healthy grandfather (Figure 4A). Notably, the expression of *SLC2A13* was similar to his grandfather (Figure 4B). We observed high levels of variation in *CNTN1* transcripts between the patient, his mother and his grandfather.

### 4.5. Transcripts Levels of LRRK2 and MUC19 in the Brain and Other Tissues

We determined the levels of *LRRK2* and *MUC19* transcripts in different regions of the brain and other major human tissues. Compared to skeletal muscle, *LRRK2* transcripts were ~14-fold higher in whole human brain, 10-fold higher in the hippocampus and cerebral cortex, and five-fold higher in the cerebellum and liver (Figure 4C). No *MUC19* transcripts were detected in tissues assayed, except in lymphocytes, where they were very low (Figure 4D).

### 4.6. Transcript Levels of HDHD1 and PNPLA4 in the Brain and Other Tissues

In the case of *HDHD1*, relative to lymphocyte, transcript levels were 12-fold higher in skeletal muscle, eight-fold higher in the fetal brain and five-fold higher in the whole human brain (Figure 4E). High levels of *PNPLA4* transcripts were detected in skeletal muscle (~70-fold increase) and the whole human brain (50-fold increase compared to lymphocytes), while levels were lower in the fetal brain, heart, kidney and liver (Figure 4F).

### 4.7. Inheritance of 12q12 and Xp22.31 Microdeletions in the DGDP289 Family

**Figure 5:**
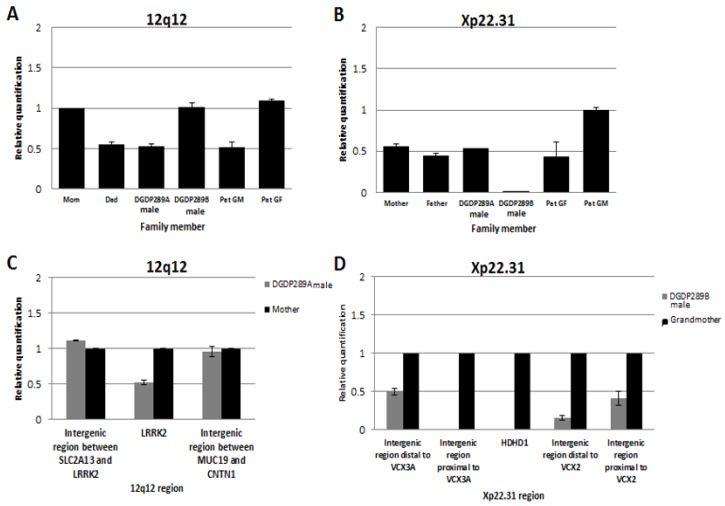
Inheritance of microdeletions in the family of DGDP289 and the refining of breakpoints by qPCR. (**A**) The 12q12 microdeletion in DGDP289A is inherited from his father, who in turn acquired it from DGDP289’s paternal grandmother (GM). All three affected family members have only one *LRRK2* allele. The patient’s mother, brother (DGDP289B) and paternal grandfather (GF) are normal, having both *LRRK2* alleles. (**B**) DGDP289B inherited the Xp22.31 microdeletion from his mother, who has only one *HDHD1* allele due to hemizygosity. DGDP289A, his father and paternal grandfather have one copy of *HDHD1* as males, because only one X-chromosome is present. The paternal grandmother is normal, with two *HDHD1* alleles. (**C**) Refining of deletion breakpoints revealed that the proximal and distal breakpoints of the 12q12 microdeletion do not extend into *SLC2A13* or *CNTN1*, respectively. (**D**) The distal breakpoint of the Xp22.31 microdeletion was found to reside either within or in the immediate telomeric vicinity of *VCX3A*. The proximal breakpoint resides within the *VCX2* gene or its centromeric proximity. Due to the repetitive nature of the VCX family, it was not possible to determine whether *VCX3A* and *VCX2* are truncated.

Assays by qPCR revealed that the 12q12 microdeletion in DGDP289A was inherited from his father, who in turn acquired it from his mother (the patient’s paternal grandmother) (Figure 5A). The mother and paternal grandfather of DGDP289A did not have the 12q12 microdeletion. qPCR assays also revealed that the Xp22.31 microdeletion in DGDP289B was inherited from his healthy mother (Figure 5B). Males are hemizygous for the Xp22.31 region, as seen in the copy number in the patient’s father compared to the paternal grandmother.

### 4.8. Delineation of Deletion Breakpoints by qPCR in DGDP289A and DGDP289B

Assays by qPCR revealed that the 12q12 microdeletion does not extend into the two neighboring genes *SLC2A13* or *CNTN1* in the centromeric and telomeric directions, respectively. The proximal breakpoint was found to reside in the intergenic region between *SLC2A13* and *LRRK2*, while the distal deletion breakpoint was located in the intergenic region between *MUC19* and *CNTN1* (Figure 5C). As a result, this microdeletion encompasses only two genes, *LRRK2* and *MUC19* at 12q12 in DGDP289A. The distal breakpoint of the Xp22.31 microdeletion was found to reside either within or in the immediate telomeric vicinity of *VCX3A*. A first intergenic region, which is 2.4 kb proximal to *VCX3A,* was deleted, while a second intergenic region, 5.7 kb distal to *VCX3A,* was intact. On the centromeric side, a 3.7 kb intergenic region distal to *VCX2* was deleted, but a 0.1 kb proximal intergenic region was intact, indicating the distal breakpoint was located between *VCX2* and *VCX3B* (Figure 5D). Because of the repetitive sequences within *VCX* genes, it was not possible to determine whether *VCX3A* and *VCX2* are truncated. At least five genes (*HDHD1*, *STS*, *VCX*, *PNPLA4*, *MIR651*) are deleted at Xp22.31 in DGDP289B.

### 4.9. LRRK2 Interacts with Proteins Previously Linked to Intellectual Disability

**Figure 6:**
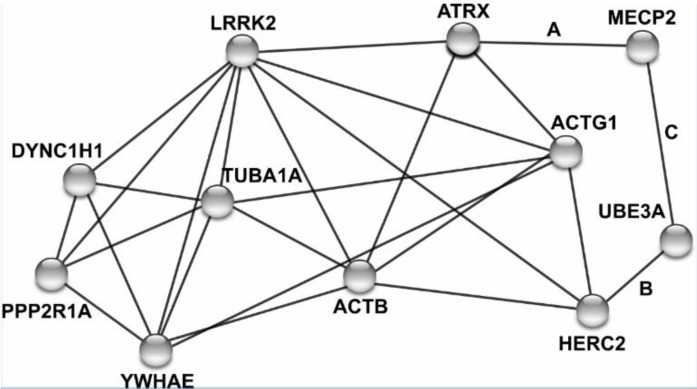
Network of LRRK2 and eight interacting proteins linked to intellectual disability. Human protein names are adjacent to nodes, with edges linking nodes based on physical interactions, co-expression, or other experimental evidence. All nodes connected to LRRK2 mark a physical protein–protein interaction. Letters A–C next to edges mark associations identified via BioGRID and a primary literature search but were not initially included in the STRING network [33,34,35].

After consolidating intellectual disability gene lists from two databases and one recent review article, we identified eight LRRK2 interacting proteins that have previously been linked to intellectual disability: ACTB [48], ACTG1 [48], ATRX [49], DYNC1H1 [50], HERC2 [51], PPP2R1A [52], TUBA1A [53,54], and YWHAE [55] (Figure 6 and Table 3). 

Expanding the network to include a secondary set of physical interactions revealed MECP2 and UBE3A interact themselves, and physically interacted with ATRX and HERC2, respectively [33,34,35]. Pathway analysis with the eight initial genes of interest with LRRK2 did not yield enrichment for notable biological pathways, however, it did identify a large proportion of the six interacting proteins that localize to the cytoskeleton (ACTB, ACTG1, DYNC1H1, HERC2, PPP2R1A, and TUBA1A).

## 5. Discussion

There are few published cases of pathogenic CNVs in the gene-rich 12q12 interval [13,14,15,18]. Patient DGDP289A displays developmental delays, autism, impaired motor skills and craniofacial anomalies and has a ~328 kb minimal microdeletion at 12q12, encompassing *LRRK2* and *MUC19* (Figure 2). We searched the DECIPHER database and identified three additional small-sized microdeletions (DCP261503, DCP255553, and DCP256710) and one microduplication DCP262066 involving *LRRK2* and *MUC19*. *LRRK2* is likely dysregulated in DCP275780 by a position effect (Figure 2) [64]. Phenotypic availability from two of the six cases shows that both DCP255553 and DCP256710 present with intellectual disability as well as craniofacial anomalies, and DCP256710 also exhibits autistic behavior (Table 4). The *MUC19* gene is involved in the synthesis of mucus, which is important for the protection of mammalian epithelia of various tissues against environmental damage. It is expressed in several glandular tissues, including the submandibular gland, submucosal gland and sublingual gland [38]. *LRRK2* is known to cause autosomal-dominant late-onset Parkinson disease 8 (PARK8, MIM 607060) [65]. It physically interacts with Parkin at 6q26 (PARK2, MIM 602544), a component of a multiprotein E3 ubiquitin ligase complex, and is the cause of 50% of autosomal recessive juvenile Parkinsonism (ARJP) [66,67,68]. The neurological function of LRRK2, along with the results of comparative deletion mapping, suggest that this gene is likely to be involved in intellectual disability, craniofacial anomalies, and autism.

The 328 kb deletion at 12q12, segregating with all three affected members of the three generations (Figure 1A), suggests the pathogenicity of this CNV in intellectual disability. In addition to depression, the father of DGDP298A has a learning disability in mathematics. This cognitive phenotype was seen in other patients with intellectual disabilities [8,69]. To further support our hypothesis that *LRRK2* is a candidate gene for cognitive impairment, we examined the transcript levels of both *LRRK2* and *MUC19* in human tissues. No *MUC19* transcripts were detected in the whole human brain, fetal brain, heart, kidney, liver and lung, while low levels of expression were recorded in lymphocytes. This observed expression pattern indeed shows that *MUC19* is highly unlikely to be the causative gene for intellectual disability and autism. Interestingly, *LRRK2* transcripts were expressed at high levels in the whole human brain, cerebral cortex and hippocampus (Figure 4C), the latter being a brain region known to be associated with memory and learning [70]. Lower expression levels of *LRRK2* were detected in the cerebellum and liver, while its transcripts were present at a much lower level in the heart, kidney, lung, and skeletal muscle. The high level of *LRRK2* expression in the different regions of the brain, particularly in the hippocampus, is consistent with a potential role in autism and learning disability. This notion is further corroborated by the clinical features of intellectual disability and autism seen in DCP255553 and DCP256710 (Figure 2 and Table 4).

The age of onset of autosomal-dominant Parkinson disease (PD), caused by *LRRK2*, is approximately 53 years [65], while autism was observed in DGDP289A at age two. This suggests that neuronal dysfunction occurring as a result of the *LRRK2* heterozygous deletion manifests early in infancy. In contrast to the gain-of-function mechanism involved in LRRK2-mediated autosomal-dominant PD [37], intellectual disability or autism appears to be due to LRRK2 loss-of-function from haploinsufficiency. Transcript levels of *SLC2A13* in DGDP289A were similar to his healthy grandfather (Figure 4B), while *CNTN1* showed a high variation in transcripts between family members (data not shown). Therefore, *SLC2A13* and *CNTN1*, flanking the microdeletion, appear unlikely to be dysregulated by a position effect.

The transcript level of *LRRK2* was drastically reduced in our microdeletion patient DGDP289A compared to his grandfather, corroborating its role in cognitive development (Figure 4A). The decreased expression of *LRRK2* in patient DGDP289A may adversely affect LRRK2 in vivo activity via decreased binding to LRRK2 binding partners. The physical interaction between LRRK2 and eight proteins with documented mutations linked to intellectual disability is further evidence to support the role of LRRK2 in cognitive impairment (Table 3). Mutations in the eight proteins listed have been shown to result in varying degrees of craniofacial abnormalities, intellectual disability, and motor or psychomotor retardation [56,57,58,59,60,61,71,72,73,74,75]. However, the relationship between LRRK2 and the eight proteins of interest have been largely studied in relation to PD or Crohn’s disease, but not intellectual disability, though extrapolations can be made. For example, LRRK2 has been shown to interact with NEURL4 and HERC2, an E3 ubiquitin ligase, and together, they negatively regulate Notch signaling and increase neuronal differentiation in embryonic mouse brain [51]. Several previous studies have shown that Notch signaling is associated with learning and memory and neuronal plasticity [76]. Based on these associations, and the interaction between LRRK2 and known intellectual disability genes, it appears that LRRK2 may play a role in a core molecular pathway that governs cognitive development, though future studies are necessary.

Though pathway analysis requires a more substantial list of genes to identify enrichment for specific biological processes, the observation that the majority of the interacting proteins are localized in the cytoskeleton is noteworthy. Previous studies have shown that alterations in dendrites and dendritic spines are a hallmark of intellectual disability, and the mutations of *LRRK2* has been shown to alter dendritic morphology [77]. In particular, loss of Lrrk2 in mice contributes to a decrease in the number of striatal projection neuron spines and alters their morphology [78], and knockdown of Lrrk2 has been systematically shown to increase neurite length [79]. The expression pattern of LRRK2 in the CNS also points to its involvement in cognitive development. Lrrk2 is first expressed on postnatal day (P) 8 in rat striatum, though it is expressed in the hippocampus and cerebral cortex as well, and expression levels increase over the course of development until they remain constant during adulthood [80]. The P8 time point in rodents is characterized by increasing axonal and dendritic density that developmentally corresponds with a late third trimester human infant [79]. Thus, the increase in *LRRK2* mRNA expression during rodent development appears to coincide with increasing synaptic density and neuronal complexity, making it a likely candidate for intellectual disability, where there are known alterations in dendritic spines and neurite length.

Comparative deletion mapping with a total of 19 CNVs delimited three chromosomal segments in the 12q12 region, in addition to the region encompassing *LRRK2* (Figure 2). The second segment is represented by a previously reported microdeletion [14]. This region also overlaps with three published cases, as well as five DECIPHER cases (Figure 2) [14,15,18]. Common clinical features between these published cases include intellectual disability and craniofacial anomalies, and are also represented in a patient [14]. Failla et al. suggested *YAF2* and *PRICKLE1* as candidate genes in their patients, which were substantiated by a smaller microdeletion found in another patient with similar phenotypes [14]. Interestingly, a homozygous mutation in *PRICKLE1* (prickle homolog 1 Drosophila, aka RILP for REST/NRSF interacting LIM domain protein) causes an autosomal recessive myoclonus epilepsy-ataxia syndrome (*EPM1B*, MIM 612437) [81]. The third segment, demarcated by the deletion in DCP250361, with clinical features of intellectual disability and craniofacial anomalies, encompasses four annotated genes (Figure 2). Among them, two genes are conspicuous. PUS7L (pseudouridylate synthase 7 homolog (S. cerevisiae)-like) interacts with APP (amyloid beta A4 protein isoform a precursor, MIM 104760) [82], a well-known gene for autosomal-dominant Alzheimer disease [83]. TWF1 (twinfilin actin-binding protein 1) interacts with APP [82] and UBE2A (ubiquitin-conjugating enzyme E2A isoform 1, MIM 312180) [84]. Intriguingly, the mutations of *UBE2A* cause XLID [85]. We propose *PUS7L* and *TWF1* as likely candidate genes on the telomeric region of 12q12 for the intellectual disability and craniofacial anomalies seen in DCP250361 (Figure 2, Table 4).

The microdeletion case reported in Carlsen et al. designates the fourth segment highlighted in gray (Figure 2), and the smaller-sized microdeletion in DCP139 is completely contained within this region [13]. Both patients display intellectual disability, craniofacial and eye anomalies. Carlsen et al. suggested two candidate genes in this region, *NELL2* (NEL-like 2 chicken) and *DBX2* (brain homeobox 2) [13]. The intragenic microdeletion of DCP139 within *NELL2* strongly suggests that it is the more likely candidate for the intellectual disability seen in the patient DCP139 (Figure 2 and Table 4). A yeast two-hybrid (Y2H) screen found that NELL2 interacts physically with CACNA1A (calcium channel, voltage-dependent, P/Q type, alpha 1A subunit) [86]. Mutations of *CACNA1A* were reported in familial hemiplegic migraine and episodic ataxia type-2, spinocerebellar ataxia type 6 (SCA6), and cognitive impairment, including intellectual disability, autism, epilepsy, and ADHD [87,88,89]. Moreover, the same study showed that NELL2 interacts with ATAXIN7 (ATXN7, MIM 607640), the causative gene for spinocerebellar ataxia type 7 (SCA7) [90]. These data suggest the plausible candidacy of *NELL2* in intellectual disability and craniofacial anomalies. As a transcription repressor, Xenopus Dbx2 (developing brain homeobox 2) is involved in primary neurogenesis and early neural plate patterning [91]. A homozygous intronic splice-acceptor mutation in *ANO6* (Anoctamin 6) resulting in a premature stop codon has been identified in a patient with Scott syndrome, a bleeding disorder defined by reduced surface exposure of procoagulant phosphatidylserine (PS) on blood cells as a consequence of activation with Ca^2+^-elevating agents [92]. Genital anomalies were also observed in patients with genomic deletions in the 12q12 interval [15,18]. However, this phenotype appears to be sporadic, displayed by patients with CNVs involving one or more of the delimited chromosomal segments (Table 4).

CNVs at Xp22.31 encompassing *STS* and *VCX3A* are frequently associated with ichthyosis, and sometimes intellectual disability [19,23,24,93]. There are at least 100 XLID genes identified so far [94,95]. Among them, *NLGN4* is the only gene identified for XLID, along with autism and Asperger syndrome at Xp22.31 [96,97] and, more than a decade ago, *VCX3A* was proposed as a candidate gene to account for intellectual disability in this region [20]. The *VCX3A* gene was found to be deleted in patients with intellectual disability, whereas patients with more proximal CNVs, not including *VCX3A,* displayed normal intelligence [20]. The manifestation of intellectual disability in individuals with CNVs encompassing *VCX3A* appears to be under the influence of incomplete penetrance, because not all male patients with a deletion involving *VCX3A* display cognitive impairment [98,99]. Four small microduplications (DCP251863, DCP256781, DCP283561, DCP255300), one microdeletion DCP1719, and microduplication Patient 7 of Esplin [42] were identified as encompassing both *HDHD1* and *STS* only. The common phenotype of all these patients is intellectual disability, strongly suggesting *HDHD1* as a candidate gene for this cognitive impairment, since *STS* is the causative for X-linked ichthyosis (Figure 3 and Table 5). This is more evident if we look at the two smallest CNVs, DCP251863 and DCP283561, with the common phenotype of intellectual disability. Although DCP256781 is not directly disrupting *HDHD1*, it is likely that a position effect dysregulates its expression. Patient 2 of Esplin [42] and three CNV patients from Decipher (DCP280938, DCP250671, and DCP251340) involving *HDHD1*, *STS*, *VCX*, *PNPLA4* also display intellectual disability. Interestingly, Patient 9 of Esplin et al., with only *PNPLA4* and *VCX* duplicated, presents with cognitive impairment. This suggests that additional genes for intellectual disability reside in the centromeric Xp22.31 region.

The transcript levels of two positional candidate genes (*HDHD1* and *PNPLA4*) in this chromosomal region were examined in the human brain and other tissues. The *HDHD1* gene is a pseudouridine-5′-phosphatase [44], while the *PNPLA4* (patatin-like phospholipase domain containing 4) encodes a triacylglycerol lipase/acetylase [47]. The transcript levels of *HDHD1* were higher in the skeletal muscle, whole human brain, and fetal brain, with significantly lower levels detected in the heart, kidney, lung and lymphocyte (Figure 4E). The high *HDHD1* expression in the brain and skeletal muscle supports its role in cognitive and muscle dysfunction, such as intellectual disability and hypotonia, exemplified in Patient 7 of Esplin et al. [42] (Figure 3 and Table 5). Transcripts levels of the second positional candidate gene (*PNPLA4*) showed a similar pattern of expression, except that its expression level is higher in the whole human brain than the fetal brain. *PNPLA4* transcript levels were lower in the heart, kidney and liver, and extremely low in the lung and lymphocytes (Figure 4F). The very high levels of *PNPLA4* transcript in the brain and skeletal muscle suggest that it also contributes to cognitive and muscle impairment, exemplified in Patient 9 of Esplin et al. [42] (Figure 3 and Table 5). 

## 6. Conclusions

We provide supporting data of the pathogenicity of the 12q12 and Xp22.31 microdeletions in neurological phenotype through comparative deletion mapping and expression analysis. Most importantly, for the first time, we show the likely involvement of LRRK2 haploinsufficiency in early-onset cognitive impairment, in stark contrast to late-onset Parkinsonism by the gain-of function mechanism of LRRK2. Additionally, we suggest *PUS7L* and *TWF1* as candidate genes for intellectual disability and craniofacial anomalies on the telomeric region of 12q12. We also propose *HDHD1* and *PNPLA4* as likely genes for XLID at Xp22.31. Further refinement of phenotype–genotype correlations at 12q12 and Xp22.31 will require the identification of smaller informative microdeletions, microduplications or rare point mutations of individual genes within these two chromosome regions. The unavailability of deleterious mutations in our positional candidate genes, including *LRRK2*, is the current limitation to this study to further investigate their functional consequences. Point mutations in single genes would be invaluable in understanding the individual contributions of each gene in syndromic intellectual disability or autism at 12q12 and Xp22.31.

## Figures and Tables

**Table 1:** Genes involved in DGDP289A with 12q12 microdeletion.

Gene	Gene Symbol	OMIM #	Remarks
Leucin-rich repeat kinase 2	*LRRK2*	609007	Interacts with Parkin and is associated with Parkinson’s Disease. Mutant LRRK2 induces neuronal degeneration [36,37].
Mucin 19	*MUC19*	612170	MUC19 is a gel-forming mucin expressed predominantly in mucous cells of various glandular tissues [38]. *MUC19* is excluded as a candidate gene because of its expression pattern.

#, denotes number.

**Table 2:** Genes involved in Xp22.31 microdeletion.

Gene	Gene Symbol	OMIM #	Remarks
Variably charged, X Chromosome 3A	*VCX3A*	300533	*VCX3A* is associated with intellectual disability [20,22].
Haloacid dehalogenase-like hydrolase domain containing 1A	*HDHD1*	306480	HDHD1 dephosporylate pseudouridine 5’-phosphate [44,45]. Expressed at higher levels in the human brain, fetal brain and skeletal muscle.
Steroid Sulfatase	*STS*	300747	*STS* is causative for X-linked ichthyosis, a skin disorder. It also has important roles in placental production of estriol during the later stages of pregnancy [23,46].
Variably charge X chromosome	*VCX*	300229	*VCX* gene appears to be expressed only in male germ cells [25].
Patatin-like phospholinasedomain containing 4	*PNPLA4*	300102	PNPLA4 highly expressed in brain and skeletal muscle. It has both triacylglycerol lipase and may be involved in adipocyte triglyceride homeostasis [47].

#, denotes number.

**Table 3:** List of LRRK2 binding partners associated with intellectual disability.

Protein Abbreviation	Protein Name	Disease Name	OMIM Phenotype #
ACTB	Actin, beta	Baraitser-Winter Syndrome 1 [56]	243310
ACTG1	Actin, gamma 1	Baraitser-Winter syndrome 2 [56]	614583
ATRX	ATRX, chromatin remodeler	(1) Alpha-thalassemia/mental retardation syndrome [57]; (2) intellectual disability-hypotonic facies syndrome, X-linked [58]	301040 309580
DYNC1H1	Dynein cytoplasmic 1 heavy chain 1	intellectual disability, autosomal dominant 13 [59]	614563
HERC2	HECT and RLD domain containing E3 ubiquitin protein ligase 2	intellectual disability, autosomal recessive 38 [60]	615516
PPP2R1A	Protein phosphatase 2 scaffold subunit alpha	intellectual disability, autosomal dominant 36 [61]	616362
TUBA1A	Tubulin alpha 1a	Lissencephaly 3 [62]	611603
YWHAE	Tyrosine 3-monooxygenase/tryptophan 5-monooxygenase activation protein epsilon	Miller-Dieker Lissencephaly Syndrome [63]	247200

#, denotes number.

**Table 4:** Clinical features of patients with 12q12 microdeletions and microduplications.

Clinical Feature	DGDP289A	Adam 2010	Gallego 2000	Tonoki 1998	Failla 2008	Carlsen 2015	DCP255553	DCP256710	DCP263528	DCP250361	DCP139	DCP285576	DCP259419	DCP280346	DCP257543	DCP261503	DCP262066	DCP285836	DCP275780	DCP285836
DD/ID	+	+	+	+	+	+	+	+	+	+	+	+	+	+	+	n/a	n/a	n/a	n/a	n/a
Autism	+	−	−	−	−	−	n/a	+	+	n/a	n/a	n/a	n/a	n/a	+	n/a	n/a	n/a	n/a	n/a
Impaired motor skills	+	+	+	+	+	+	n/a	n/a	n/a	n/a	n/a	n/a	+	+	n/a	n/a	n/a	n/a	n/a	n/a
Craniofacial anomalies	+	+	+	+	+	+	+	+	n/a	+	+	+	n/a	n/a	n/a	n/a	n/a	n/a	n/a	n/a
Eye anomalies	−	−	+	+	+	+	n/a	n/a	+	n/a	+	+	n/a	n/a	n/a	n/a	n/a	n/a	n/a	n/a
Microcephaly	−	+	-	+	+	−	n/a	−	n/a	n/a	n/a	n/a	n/a	n/a	n/a	n/a	n/a	n/a	n/a	n/a
Small hands	−	+	+	+	-	+	n/a	n/a	n/a	n/a	n/a	n/a	n/a	n/a	n/a	n/a	n/a	n/a	n/a	n/a
Clinodactyly	−	−	+	+	+	+	n/a	n/a	n/a	n/a	n/a	n/a	n/a	n/a	n/a	n/a	n/a	n/a	n/a	n/a
Hypotonia	−	−	−	+	+	+	+	n/a	n/a	n/a	n/a	n/a	n/a	n/a	n/a	n/a	n/a	n/a	n/a	n/a
Sensorineural hearing loss	−	−	-	+	+	−	n/a	n/a	+	n/a	n/a	n/a	n/a	n/a	n/a	n/a	n/a	n/a	n/a	n/a
Cardiac anomalies	−	−	-	-	n/a	+	n/a	n/a	n/a	n/a	n/a	n/a	n/a	n/a	n/a	n/a	n/a	n/a	n/a	n/a
Genital anomalies	−	−	-	+	+	−	n/a	+	n/a	n/a	+	n/a	n/a	n/a	+	n/a	n/a	n/a	n/a	n/a

DD, denotes developmental delay; ID, represents intellectual disability; +, indicates that the clinical feature was displayed by the corresponding patient; −, shows that the clinical feature was absent; n/a, for not available.

**Table 5:** Clinical features of patients with Xp22.31 microdeletions and microduplications.

Clinical Features	DGDP289B	Hosomi 2007	Khelifa 2013	Esplin 2014 Pt 1	Esplin 2014 Pt 2	Esplin 2014 Pt 7	Esplin 2014 Pt 9	DCP251863	DCP256781	DCP283561	DCP255300	DCP1719	DCP280938	DCP250671	DCP251340
Gender	M	M	M	M	M	M	M	F	F	F	M	M	M	M	M
DD/ID	+	+	+	−	+	+	+	+	+	+	+	+	+	+	+
Ichthyosis	+	+	+	n/a	n/a	−	n/a	n/a	n/a	n/a	n/a	+	+	n/a	n/a
Craniofacial anomalies	+	−	+	−	+	+	+	n/a	n/a	n/a	n/a	n/a	+	+	n/a
Seizures	−	−	+	+	−	+	−	n/a	n/a	+	n/a	+	n/a	n/a	n/a
Sensorineural hearing impairment	−	−	−	−	−	−	−	n/a	n/a	n/a	n/a	+	n/a	n/a	n/a
Hypotonia	−	−	−	−	−	+	+	−	−	−	−	−	−	−	−

DD, denotes developmental delay; ID, represents intellectual disability; +, indicates that the clinical feature was displayed by the corresponding patient; −, shows that the clinical feature was absent; Pt, denotes patient; n/a, not available; M, Males; F. females.

## Supplementary Materials

The following are available online at https://www.mdpi.com/2077-0383/9/1/274/s1, Table S1: Primers used for qPCR and RT-qPCR.
