# Supplementary material for: Comparative Genomic Mapping Implicates LRRK2 for Intellectual Disability and Autism at 12q12, and HDHD1, as Well as PNPLA4, for X-Linked Intellectual Disability at Xp22.31
Source: J Clin Med. 2020 Jan 19;9(1):274. doi: 10.3390/jcm9010274 (PMC7019335; doi:10.3390/jcm9010274)
Supplement: Supplementary file 1 [file jcm-09-00274-s001.pdf]

Table S1. Primers used for qPCR and RT-qPCR.

Primer name	Forward primer 5′-3′	Reverse primer 5′-3′
*#LRRK2Ex24F1-R1	GAAATGACATTGGACCCTCAGTGGT	AGGTTCTCAGGTACAAAAGACAGC
#MUC19Ex7F1-R1	CAACACAAGATATGGTGCTGGCTT	GCTTTACTGACTTCCAATCCCTTT
#SLC2A13Ex2F1-R1	ATTGCTTCTATGACAGTGCCAGTG	TGTGATGAAGAGGGTATTAATGGTGA
*#HDHD13UTRF2-R2	GCAATGACAACTGATAAAGCGAGATG	GCTAACAAGGAGTGCTCATCAAAAAC
#PNPLA43UTRF1-R1	GCACATGGATATAAGAGGCACTTTAT	AGGACACAACAGTTCACCAAAAATGA
*12q12F3-R3	CCTCATTTAGGGCTCTGGCAACC	CGGCAAACATTCTGGGTGGCTTC
*12q12F6-R6	TTCACTTCAATGATGAGGACCCACTA	CCTACCCAGTTTTGTGTATGAGAGAG
*Xp22.31F10-R10	ATGAGTTTCAGGGTCAGGTTCACTTG	ACATAGGGAATCTAGTCTCGTCAAAT
*Xp22.31F11-R11	AGACTTTTCAGCCTCTAATTCCCCT	CTCCCTCACATTACCCCTATGGATT
*Xp22.31F17-R17	AGGAGAAAGATTAAGGCTGGAACACT	CACTCTGGGTGGGTAACATCAAATCA
*Xp22.31F13-R13	TGCCTCACAATTTTTCATTCTCATGG	GCCTGATACCGAAACCCAGCCTGATA

* Primers used for qPCR; # Primers used for RT-qPCR
